# Supplementary material for: Stigma towards Mental Disorders among Nursing Students and Professionals: A Bibliometric Analysis
Source: Int J Environ Res Public Health. 2022 Feb 6;19(3):1839. doi: 10.3390/ijerph19031839 (PMC8835101; doi:10.3390/ijerph19031839)
Supplement: Supplementary file 1 [file ijerph-19-01839-s001.zip › ijerph-1494487-supplementary.pdf]

Table S1: Complementary information on the retrieved articles

Authors	Title	Source	Language	Study Tipe	First author institution	Country	†GBD Regions	††Categorization SDI
(Abasiubong et al, 2007)	A comparative study of attitude to mental illness between journalists and nurses in Uyo, Nigeria.	African journal of medicine and medical sciences	English	Observational	University of Uyo. Department of Psychiatry, Faculty of Clinical Sciences, College of Health Sciences	Nigeria	Western sub-Saharan Africa	Low-middle SDI
(Abuhammad et al, 2019)	Correlates and Predictors of Stigmatization of Patients With Mental Illness Among Nursing Students	Journal of psychosocial nursing and mental health services	English	Observational	Jordan University of Science and Technology. Department of Maternal and Child Health	Jordan	North Africa and Middle East	Middle SDI
(Al-Awadhi et al, 2017)	Nurses' Attitude Towards Patients with Mental Illness in a General Hospital in Kuwait	Saudi journal of medicine & medical sciences	English	Observational	Mubarak Al-Kabeer Hospital. Department of Emergency Medicine	Kuwait	North Africa and Middle East	Middle SDI
(Alyousef et al, 2019)	Perceptions of Media's Contribution Toward Stigmatization of Mental Health by Saudi Arabian Nurses.	Journal of the American Psychiatric Nurses Association	English	Observational	King Saud University. Seham Mansour Alyousef, PhD	Saudi Arabia	North Africa and Middle East	Middle SDI
(Anandan et al, 2016)	Nursing attitudes towards people with comorbid substance abuse: a brief review of literature.	Australian nursing & midwifery journal	English	Review	Monash University	Australia	Australasia	High SDI
(Arbanas et al, 2018)	Impact of a Nursing in Psychiatry Course on Students' Attitudes Toward Mental Health Disorders	Journal of psychosocial nursing and mental health services	English	Intervention	University Psychiatric Hospital Vrapce	Croatia	Central Europe	High-middle SDI

(Ashencaen Crabtree, 2003)	Asylum blues: staff attitudes towards psychiatric nursing in Sarawak, East Malaysia.	Journal of psychiatric and mental health nursing	English	Observational	Zayed University, College of Family Sciences	United Arab Emirates	North Africa and Middle East	Middle SDI
(Avanci et al, 2002)	Autoritarismo e benevolência frente à doença mental: estudo com alunos ingressantes no curso de enfermagem	Revista Latino-Americana de Enfermagem	Portuguese	Observational	Prefeitura Municipal de Ribeirão Preto. Faculdade de educação São Luís	Brasil	Tropical Latin America	Middle SDI
(Ayuso Gutierrez et al, 1980)	A comparative study of the psychiatric nurse's attitudes toward mental patients.	International journal of rehabilitation research.	English	Observational	Medical School, Hospital Clínico, Madrid	Spain	Western Europe	High SDI
(Ayuso-Gutierrez et al, 1978)	Comparative-study of psychiatric nurses attitudes towards mental-patients	International journal of social psychiatry	English	Observational	Medical School, Hospital Clínico, Madrid	Spain	Western Europe	High SDI
(Bairan et al, 1989)	Attitudes toward mental illness: does a psychiatric nursing course make a difference?	Archives of psychiatric nursing	English	Observational	University Teknikal Malaysia Melaka	Malaysia	Southeast Asia	Middle SDI
(Baker et al, 2005)	Nursing attitudes towards acute mental health care: development of a measurement tool	Journal of advanced nursing	English	Other	University of Manchester. School of Nursing, Midwifery and Social Work	UK England	Western Europe	High SDI
(Bennett et al, 2015)	Attitudes towards mental illness of nursing students in a Baccalaureate programme in Jamaica: a questionnaire survey	Journal of psychiatric and mental health nursing	English	Observational	University of the West Indies. School of Nursing	Jamaica	Caribbean	Middle SDI
(Bergman et al, 2010)	Nurses attitudes and expectations toward psychiatric patients with multiple readmissions	Israel annals of psychiatry and related disciplines	English	Observational	Jdc Malben,Dept Med Serv & Care Aged	Israel	Western Europe	High SDI
(Berner, 2008)	Psychiatric nursing. Against stigmatization an herb has grown.	Krankenpflege. Soins infirmiers	English	Observational	Private Clinic Wyss AG	Switzerland	Western Europe	High SDI

(Bertram et al, 2005)	Mental health nurses, promoters of inclusion or perpetuators of exclusion?	Journal of psychiatric and mental health nursing	English	Observational	Nottinghamshire Healthcare NHS Trust	UK, England	Western Europe	High SDI
(Bilge et al, 2017)	The Effect of Short Films About Mental Health and Disorders on Preventing Stigmatization in Nursing Education	Archives of psychiatric nursing	English	Intervention	Ege University, Nursing Faculty, Psychiatric and Mental Health Nursing Department	Turkey	North Africa and Middle East	Middle SDI
(Bingham et al, 2018)	Educational intervention to decrease stigmatizing attitudes of undergraduate nurses towards people with mental illness	International journal of mental health nursing	English	Intervention	Western Institute of Technology. Department of Nursing	New Zealand	Australasia	High SDI
(Bjorkman et al, 2008)	Attitudes towards people with mental illness: a cross-sectional study among nursing staff in psychiatric and somatic care	Scandinavian journal of caring sciences	English	Observational	Lund University. Department of Health Sciences, Division of Nursing	Sweden	Western Europe	High SDI
(Bocking et al, 2019)	'It is meant to be heart rather than head'; International perspectives of teaching from lived experience in mental health nursing programs	International journal of mental health nursing	English	Observational	Australian National University, ANU Medical School	Australia	Australasia	High SDI
(Brady, 2003)	Nurses' attitudes towards a patient who has a psychiatric history.	Journal of advanced nursing	English	Observational	St. Vincent's Hospital School of Nursing	USA	High-income North America	High SDI
(Brinn, 2000)	Patients with mental illness: general nurses' attitudes and expectations.	Nursing standard	English	Observational	Whitchurch Hospital	UK, Wales	Western Europe	High SDI
(Burnard et al, 2006)	Views of mental illness and mental health care in Thailand: a report of an ethnographic study	Journal of psychiatric and mental health nursing	English	Observational	Cardiff University. School of Nursing and Midwifery Studies	UK, Wales	Western Europe	High SDI

(Burns et al, 2017)	What's wrong with John? a randomised controlled trial of Mental Health First Aid (MHFA) training with nursing students	Bmc psychiatry	English	Intervention	Curtin University, School of Public Health	Australia	Australasia	High SDI
(Byrne et al, 2014)	Changing Nursing Student Attitudes to Consumer Participation in Mental Health Services: A Survey Study of Traditional and Lived Experience-led Education	Issues in mental health nursing	English	Intervention	Central Queensland University. Institute for Health and Social Science Research, Centre for Mental Health Nursing Innovation and School of Nursing and Midwifery.	Australia	Australasia	High SDI
(Callaghan et al, 1996)	The effect of a psychiatric secondment on Hong Kong chinese student nurses' attitudes to mental illness.	Journal of psychiatric and mental health nursing	English	Observational	City University. Research Fellow, Department of Mental Health and Learning Disability Nursing	UK, England	Western Europe	High SDI
(Callaghan et al, 1997)	Attitudes towards mental illness: Testing the contact hypothesis among Chinese student nurses in Hong Kong	Journal of advanced nursing	English	Intervention	Chinese University of Hong Kong. Department of Nursing	Hong Kong	East Asia	High-middle SDI
(Carroll, 2018)	Destigmatizing mental illness an innovative evidence-based undergraduate curriculum	Journal of psychosocial nursing and mental health services	English	Intervention	Anna Maria Colleg	USA	High-income North America	High SDI
(Carvalho et al, 2017)	Nursing students' depiction of mental disorder	Journal of mental health training education and practice	English	Observational	Escola Superior de Enfermagem do Porto	Portugal	Western Europe	High SDI

(Cassel et al, 1970)	Comparing opinions about mental illness for hospital attendants and practical nursing students	Nursing research	English	Observational	University of British Columbia. School of Health and Exercise Sciences	Canada	High-income North America	High SDI
(Chambers et al, 2004)	Nurses' attitudes to mental illness: A comparison of a sample of nurses from five European countries	International journal of nursing studies	English	Observational	University of London and Kingston University. Faculty of Health and Social Care Sciences, St George's	UK, England	Western Europe	High SDI
(Chang et al, 2017)	Stigma towards mental illness among medical and nursing students in Singapore: a cross-sectional study	Bmj open	English	Observational	Research Division, Institute of Mental Health	Singapore	High-income Asia Pacific	High SDI
(Charleston et al, 2005)	Psychiatric nurses and undergraduate nursing students' perceptions of preceptorship in the mental health setting.	The international journal of psychiatric nursing research	English	Observational	University of Melbourne. Centre for Psychiatric Nursing Research and Practice School of Nursing	Australia	Australasia	High SDI
(Chung et al, 2008)	A Study on the Stigma held by Nursing College Students against Mental Illness and Mentally Ill Patients	Korean Journal of Social Issues	Korean	Observational	Dongshin University	South Korea	High-income Asia Pacific	High SDI
(Creech, 1977)	Changes in attitudes about mental-illness among nursing-students following a psychiatric affiliation	Journal of psychosocial nursing and mental health services	English	Observational	Center of Excellence for Research on Returning War Veterans and the Central Texas Veterans Health	USA	High-income North America	High SDI
(Curtis, 2007)	Working together: A joint initiative between academics and clinicians to prepare undergraduate nursing students to work in mental health settings	International journal of mental health nursing	English	Intervention	University of Wollongong. School of Nursing, Midwifery and Indigenous Health	Australia	Australasia	High SDI

(de Jacq et al, 2016)	The Variability of Nursing Attitudes Toward Mental Illness: An Integrative Review	Archives of psychiatric nursing	English	Review	Columbia University School of Nursing	USA	High-income North America	High SDI
(Deans et al, 2006)	Attitudes of registered psychiatric nurses towards patients diagnosed with borderline personality disorder.	Contemporary nurse	English	Observational	University of Ballarat and Ballarat Health Services, Ballarat, and North West Health Care Network, Royal Melbourne Hospital	Australia	Australasia	High SDI
(Delaney, 2012)	Psychiatric Mental Health Nurses: Stigma Issues We Fail to See	Archives of psychiatric nursing	English	Observational	Rush College of Nursing, Department of Community Mental Health and Systems	USA	High-income North America	High SDI
(Dickens et al, 2016)	Interventions to improve mental health nurses' skills, attitudes, and knowledge related to people with a diagnosis of borderline personality disorder: Systematic review	International journal of nursing studies	English	Review	Abertay University. Division of Mental Health Nursing and Counselling	UK, England	Western Europe	High SDI
(Dickens et al, 2019)	Mixed-methods evaluation of an educational intervention to change mental health nurses' attitudes to people diagnosed with borderline personality disorder	Journal of clinical nursing	English	Intervention	Abertay University. School of Health and Social Sciences	UK, Scotland	Western Europe	High SDI
(Dickens et al, 2019)	Mental health nurses' attitudes, behaviour, experience and knowledge regarding adults with a diagnosis of borderline personality disorder: systematic, integrative literature review	Journal of clinical nursing	English	Review	Abertay University. Division of Mental Health Nursing and Counselling	UK, Scotland	Western Europe	High SDI

(Dickens et al, 2019)	Mental health nurses' attitudes, experience, and knowledge regarding routine physical healthcare: systematic, integrative review of studies involving 7,549 nurses working in mental health settings	Bmc nursing	English	Review	Western Sydney University.Centre for Applied Nursing Research (CANR)	Australia	Australasia	High SDI
(Dixon, 2003)	A nursing student's impression of mental illness.	Journal of psychiatric and mental health nursing	English	Observational	Khoy University of Medical Sciences. Department of Medical-Surgical Nursing, School of Nursing,	Iran	North Africa and Middle East	Middle SDI
(Douglas et al, 2018)	Nurses' perception of preparedness for moving mental health care from psychiatric to general hospitals in Jamaica.	Revista panamericana de salud publica = Pan American journal of public health	English	Observational	University of the West Indies Mona Campus. Department of Community Health and Psychiatry, Faculty of Medical Sciences	Jamaica	Caribbean	Middle SDI
(Duman et al, 2017)	Effects of two different psychiatric nursing courses on nursing students' attitudes towards mental illness, perceptions of psychiatric nursing, and career choices	Journal of professional nursing	English	Intervention	Dokuz Eylul University. Faculty of Nursing, Psychiatric Nursing Department	Turkey	North Africa and Middle East	Middle SDI
(Ebrahimi et al, 2017)	Comparing Mental Illness Stigma among Nurses in Psychiatric and Non-Psychiatric Wards in Tabriz University of Medical Sciences	Acta facultatis medicae naissensis	English	Observational	Tabriz University of Medical Sciences. Department of Psychiatric Nursing, Nursing and Midwifery Faculty	Iran	North Africa and Middle East	Middle SDI
(Eisenmar, 1972)	Creativity in student nurses and their attitudes toward mental-illness and physical disability	Journal of clinical psychology	English	Other	University of Texas	USA	High-income North America	High SDI

(Enshi et al, 1978)	The current problems of public health nurses at Ishikawa Pref. Improved mental hygiene and public health nursing: a change in the attitude of a public health nurse toward mental patients].	Hokenfu zasshi The Japanese journal for public health nurse	English	Observational	Graduate School of Biomedical Sciences. Department of Obstetrics and Gynecology	Japan	High-income Asia Pacific	High SDI
(Ewalds-Kvist et al, 2013)	Student nurses and the general population in Sweden: Trends in attitudes towards mental illness	Nordic journal of psychiatry	English	Observational	Stockholm University. Department of Psychology	Sweden	Western Europe	High SDI
(Farley-Toombs, 2012)	The Stigma of a Psychiatric Diagnosis: Prevalence, Implications and Nursing Interventions in Clinical Care Settings	Critical care nursing clinics of north america	English	Review	Strong Memorial Hospital, Rochester	USA	High-income North America	High SDI
(Fernandes et al, 2019)	Opinions on mental illness from the perspective of primary care nurses in Portugal.	Revista gaucha de enfermagem	English	Observational	Universidade Fernando Pessoa (UFP), Escola Superior de Saúde. Porto	Portugal	Western Europe	High SDI
(Fokuo et al, 2017)	Decreasing the Stigma of Mental Illness Through a Student-Nurse Mentoring Program: A Qualitative Study	Community mental health journal	English	Intervention	Illinois Institute of Technology	USA	High-income North America	High SDI
(Fokuo et al, 2019)	Pilot of a consumer based anti-stigma mentorship program for nursing students	Journal of public mental health	English	Intervention	University of California. Department of Psychiatry	USA	High-income North America	High SDI
(Foster et al, 2019)	Undergraduate nursing students' stigma and recovery attitudes during mental health clinical placement: A pre/post-test survey study	International journal of mental health nursing	English	Observational	Australian Catholic University, School of Nursing, Midwifery and Paramedicine	Australia	Australasia	High SDI

(FuentesOlavarría et al, 2019)	Change of attitude by Nursing students towards mental illnesses through experiential learning	Educación médica superior	Spanish	Intervention	Universidad Del Desarrollo, Facultad de Medicina Clínica Alemana	Chile	Southern Latin America	High-middle SDI
(Gandhi et al, 2019)	Knowledge and perceptions of Indian primary care nurses towards mental illness	Investigación y Educación en Enfermería	English	Observational	Deemed University. Department of Nursing, National Institute of Mental Health & Neurosciences	India	South Asia	Low-middle SDI
(Gandhi et al, 2019b)	Knowledge and perceptions of Indian primary care nurses towards mental illness.	Investigacion y educacion en enfermeria	English	Observational	Deemed University.Department of Nursing, National Institute of Mental Health & Neurosciences	India	South Asia	Low-middle SDI
(Goodwin et al, 2008)	Psychiatric nurses' attitudes toward consumer and carer participation in care: part 2--barriers to participation.	Policy, politics & nursing practice	English	Observational	Victoria University	Australia	Australasia	High SDI
(Gouthro, 2009)	Recognizing and addressing the stigma associated with mental health nursing: a critical perspective.	Issues in mental health nursing	English	Observational	University of Jordan. Department of Community Health Nursing, Faculty of Nursing	Jordan	North Africa and Middle East	Middle SDI
(Granados-Gamez et al, 2017)	Attitudes and Beliefs of Nursing Students Toward Mental Disorder: The Significance of Direct Experience With Patients	Perspectives in psychiatric care	English	Observational	University of Almeria. Faculty of Health Sciences	Spain	Western Europe	High SDI
(Gray et al, 2017)	What does mental health nursing contribute to improving the physical health of service users with severe mental illness? A thematic analysis	International journal of mental health nursing	English	Observational	Hamad Medical Corporation, Doha	Qatar	North Africa and Middle East	Middle SDI

(Grover et al, 2019)	Change in Attitude among Nursing Undergraduate Students Following One-Month Exposure in a Mental Healthcare Setting	Indian journal of psychology medicine	English	Observational	Institute of Human Behaviour and Allied Sciences. Department of Clinical Psychology	India	South Asia	Low-middle SDI
(Haddad et al, 2007)	District nursing staff and depression: A psychometric evaluation of Depression Attitude Questionnaire findings	International journal of nursing studies	English	Observational	King's College London, Institute of Psychiatry. Section of Psychiatric Nursing	UK, England	Western Europe	High SDI
(Haddad et al, 2010)	School nurses' involvement, attitudes and training needs for mental health work: a UK-wide cross-sectional study	Journal of advanced nursing	English	Observational	Institute of Psychiatry at King's College London. Health Service and Population Research Department	UK, England	Western Europe	High SDI
(Halter, 2004)	Stigma and help seeking related to depression: a study of nursing students.	Journal of psychosocial nursing and mental health services	English	Observational	Malone College	USA	High-income North America	High SDI
(Halter, 2008)	Perceived characteristics of psychiatric nurses: Stigma by association	Archives of psychiatric nursing	English	Observational	University of Akron, College of Nursing	USA	High-income North America	High SDI
(Hamdan-Mansour et al, 2009)	Attitudes of Jordanian mental health nurses toward mental illness and patients with mental illness.	Issues in mental health nursing	English	Observational	University of Jordan. Department of Community Health Nursing, Faculty of Nursing,	Jordan	North Africa and Middle East	Middle SDI
(Hanzawa et al, 2012)	Study of understanding the internalized stigma of schizophrenia in psychiatric nurses in Japan	Psychiatry and clinical neurosciences	English	Observational	Jichi Medical University. School of Nursing	Japan	High-income Asia Pacific	High SDI

(Happell et al, 2007)	Undergraduate nursing students' attitudes towards mental health nursing: Determining the influencing factors	Contemporary nurse	English	Observational	Central Queensland University. Department of Nursing and Health Studies	Australia	Australasia	High SDI
(Happell et al, 2008)	Developing more positive attitudes towards mental health nursing in undergraduate students: part 1 - does more theory help?	Journal of psychiatric and mental health nursing	English	Observational	Central Queensland University. Department of Health Innovation and CQU Healthy Communities	Australia	Australasia	High SDI
(Happell et al, 2009a)	Nursing Students' Attitudes to Mental Health Nursing: Psychometric Properties of a Self-report Scale	Archives of psychiatric nursing	English	Observational	University Australia. Department of Health Innovation	Australia	Australasia	High SDI
(Happell et al, 2014)	Lived-experience participation in nurse education: Reducing stigma and enhancing popularity	International journal of mental health nursing	English	Intervention	Central Queensland University, Institute for Health and Social Science Research, Centre for Mental Health Nursing Innovation	Australia	Australasia	High SDI
(Happell et al, 2015)	Lived experience in teaching mental health nursing: Issues of fear and power	International journal of mental health nursing	English	Observational	University of Canberra. Research Centre for Nursing and Midwifery Practice, Faculty and Health, and ACT Health,	Australia	Australasia	High SDI
(Happell et al, 2018)	Nursing Students' Attitudes Towards People Diagnosed with Mental Illness and Mental Health Nursing: An international Project from Europe and Australia	Issues in mental health nursing	English	Observational	University of Newcastle, School of Nursing and Midwifery	Australia	Australasia	High SDI

(Happell et al, 2019a)	Nursing student attitudes to people labelled with 'mental illness' and consumer participation: A survey-based analysis of findings and psychometric properties	Nurse education today	English	Observational	University of Newcastle, School of Nursing and Midwifery	Australia	Australasia	High SDI
(Happell et al, 2019b)	Changing attitudes: The impact of Expert by Experience involvement in Mental Health Nursing Education: An international survey study	International journal of mental health nursing	English	Observational	University of Newcastle, School of Nursing and Midwifery	Australia	Australasia	High SDI
(Happell et al, 2019c)	'There's more to a person than what's in front of you': Nursing students' experiences of consumer taught mental health education	International journal of mental health nursing	English	Observational	University of Newcastle. School of Nursing and Midwifery,	Australia	Australasia	High SDI
(Happell et al, 2019d)	I felt some prejudice in the back of my head: Nursing students' perspectives on learning about mental health from "Experts by Experience"	Journal of psychiatric and mental health nursing	English	Observational	University of Newcastle, School of Nursing and Midwifery	Australia	Australasia	High SDI
(Happell, 2005)	Mental Health Nursing: Challenging stigma and discrimination towards people experiencing a mental illness	International journal of mental health nursing	English	Other	University of Melbourne, School of Nursing	Australia	Australasia	High SDI
(Happell, 2008a)	The importance of clinical experience for mental health nursing - Part 1: Undergraduate nursing students' attitudes, preparedness and satisfaction	International journal of mental health nursing	English	Observational	Central Queensland University. Contemporary Nursing, Department of Nursing and Health Studies	Australia	Australasia	High SDI

(Happell, 2008b)	The importance of clinical experience for mental health nursing - Part 2: Relationships between undergraduate nursing students' attitudes, preparedness, and satisfaction	International journal of mental health nursing	English	Observational	Central Queensland University. Department of Health Innovation and Centre for Social Science	Australia	Australasia	High SDI
(Happell, 2009b)	Influencing undergraduate nursing students' attitudes toward mental health nursing: acknowledging the role of theory.	Issues in mental health nursing	English	Observational	Central Queensland University	Australia	Australasia	High SDI
(Hayman-White et al, 2005)	Nursing students' attitudes toward mental health nursina and consumers: Psychometric properties of a self-report scale	Archives of psychiatric nursing	English	Observational	University of Melbourne. School of Nursing, Faculty of Medicine, Dentistry and Health Sciences, Centre for Psychiatric Nursing Research and Practice	Australia	Australasia	High SDI
(Heim et al, 2019)	Reducing mental health-related stigma among medical and nursing students in low- and middle-income countries: a systematic review.	Epidemiology and psychiatric sciences	English	Review	University of Zurich. Department of Psychology	Switzerland	Western Europe	High SDI
(Hellzen et al, 2018)	Nurses' attitudes towards older residents with long-term schizophrenia	Journal of advanced nursing	English	Observational	Mid-Sweden University. Department of Health and Caring Science,	Sweden	Western Europe	High SDI
(Hogberg et al, 2008)	Attitudes towards mental illness in Sweden: Adaptation and development of the Community Attitudes towards Mental Illness questionnaire	International journal of mental health nursing	English	Observational	Karolinska Institute. Division of Psychiatry, Institution of Clinical Neuroscience	Sweden	Western Europe	High SDI

(Hsiao et al, 2015)	Factors influencing mental health nurses' attitudes towards people with mental illness	International journal of mental health nursing	English	Observational	Chung Shan Medical University, School of Nursing, College of Medicineç	Taiwan	East Asia	High-middle SDI
(Hugo, 2001)	Mental health professionals' attitudes towards people who have experienced a mental health disorder.	Journal of psychiatric and mental health nursing	English	Observational	North-western Adelaide Mental Health Service, Queen Elizabeth Hospital	Australia	Australasia	High SDI
(Hunter et al, 2015)	Nursing Students ' Attitudes about Psychiatric Mental Health Nursing	Issues in mental health nursing	English	Observational	Kings College. Institute of Psychiatry	UK, England	Western Europe	High SDI
(Ihalainen-Tamlander et al, 2016)	Stigmatizing attitudes in nurses towards people with mental illness: a cross-sectional study in primary settings in Finland	Journal of psychiatric and mental health nursing	English	Observational	University of Turku. Department of Nursing Science	Finland	Western Europe	High SDI
(Iheanacho et al, 2014)	Attitudes Toward Mental Illness and Changes Associated with a Brief Educational Intervention for Medical and Nursing Students in Nigeria	Academic psychiatry	English	Intervention	Yale University School of Medicine	USA	High-income North America	High SDI
(Inan et al, 2019)	The Impact of Mental Health Nursing Module, Clinical Practice and an Anti-Stigma Program on Nursing Students' Attitudes toward Mental Illness: A Quasi-Experimental Study	Journal of professional nursing	English	Intervention	Dokuz Eylül University, Nursing Faculty, Department of Psychiatric Nursing	Turkey	North Africa and Middle East	Middle SDI

(Itzhaki et al, 2017)	Nursing Students' Attitudes and Intention to Work with Mentally Ill Patients Before and After a Planned Intervention	Academic psychiatry	English	Intervention	Tel Aviv University	Israel	Western Europe	High SDI
(James et al, 2007)	Psychiatric nurses' knowledge, experience and attitudes towards clients with borderline personality disorder	Journal of psychiatric and mental health nursing	English	Observational	Royal College of Surgeons in Ireland, and Youth Drug & Alcohol Service (YoDA), Tallaght	UK, Ireland	Western Europe	High SDI
(Jang Mi-yount et al, 2017)	Care Burden for Mental Illness Patients, Attitude toward Mental Illness and Psychiatric Nursing Competency in Non-psychiatric Nurses	Journal of Korean Academy of Psychiatric and Mental Health Nursing	Korean	Observational	Kyung Hee University. College of Nursing Science	South Korea	High-income Asia Pacific	High SDI
(Jarvis, 2006)	District nurses' involvement and attitudes to mental health problems: A three-area cross-sectional study.	Journal of clinical nursing	English	Other	Inst Psychiat, Hlth Serv Res Dept, Sect Psychiat Nursing	UK, Scotland	Western Europe	High SDI
(Julia-Sanchis et al, 2019)	The Spanish version of the Psychiatric/Mental health clinical placement survey and an assessment of Spanish student attitudes towards mental health	Journal of psychiatric and mental health nursing	English	Observational	University of Alicante. Nursing Department, Health Sciences Faculty	Spain	Western Europe	High SDI
(Kahn, 1976)	Relationship between nurses' opinions about mental illness and experience.	Nursing research	English	Observational	California State University	USA	High-income North America	High SDI
(Katsuki et al, 2005)	A study of emotional attitude of psychiatric nurses: Reliability and validity of the Nurse Attitude Scale	International journal of menta health nursing	English	Observational	Niigata University. School of Health Sciences, Faculty of Medicine	Japan	High-income Asia Pacific	High SDI

(Keane, 1991)	Beliefs about mental illness in a culturally diverse nursing student population: implications for education and practice.	The Journal of the New York State Nurses' Association	English	Observational	City University of New York, City College School of Nursing	USA	High-income North America	High SDI
(Khorasanizadeh et al, 2018)	A comparative survey of the attitudes of nurses, nursing students, and patients as to the observance of the patients' dignity in the psychiatric ward	Australasian medical journal	English	Observational	Shahid Beheshti University of Medical Sciences. School of Nursing & Midiwifery	Iran	North Africa and Middle East	Middle SDI
(Kim et al, 1978)	A study on knowledge and attitudes toward mental illness and psychiatric nursing needs of Korean people.	Taehan kanho. The Korean nurse	Japanese	Observational	Yonsei University	South Korea	High-income Asia Pacific	High SDI
(Kim et al, 2016)	The Prejudice towards the Mental Illness according to Clinical Practice Experience of Community Mental Health among Nursing Students	Asia-pacific Journal of Multimedia Services Convergent with Art, Humanities, and Sociology	Korean	Observational	Busan Catholic University	South Korea	High-income Asia Pacific	High SDI
(Kirkpatrick, 2008)	A narrative framework for understanding experiences of people with severe mental illnesses	archives of psychiatric nursing	English	Observational	Hamilton and McMaster University. St. Joseph's Healthcare	Canada	High-income North America	High SDI
(Koukia et al, 2013)	Greek Mental Health Nurses' Practices and Attitudes in the Management of Acute Cases	Issues in mental health nursing	English	Observational	University of Athens, Faculty of Nursing	Greece	Western Europe	High SDI
(Kumakura et al, 1992)	Attitude change towards mental illness during nursing education--a cross-cultural study of student nurses in Korea, Republic of	Asia-Pacific journal of public health	English	Observational	Toho University. Department of Mental Health, School of Health Sciences	Japan	High-income Asia Pacific	High SDI

	China and Japan.							
(Lee et al, 2018)	The Attitudes toward Mental Illness in Nursing Students after Clinical Practice of Psychiatric Nursing	Asia-pacific Journal of Multimedia Services Convergent with Art, Humanities, and Sociology	Korean	Intervention	Department of Mental Health, School of Health Sciences, Tokyo University	South Korea	High-income Asia Pacific	High SDI
(Lethoba et al, 2006)	How professional nurses in a general hospital setting perceive mentally ill patients.	Curationis	English	Observational	UNISA. Universidade Santo Amaro	Brasil	Tropical Latin America	Middle SDI
(Linden et al, 2012)	Attitudes of qualified vs. student mental health nurses towards an individual diagnosed with schizophrenia	Journal of advanced nursing	English	Observational	Queen's University of Belfast. School of Nursing & Midwifery	UK, Ireland	Western Europe	High SDI
(Madianos et al, 2005)	Nursing students' attitude change towards mental illness and psychiatric case recognition after a clerkship in psychiatry.	Issues in mental health nursing	English	Observational	University of Athens. School of Health Sciences, Faculty of Nursing	Greece	Western Europe	High SDI
(Malla et al, 1987)	Attitudes towards mental illness: the influence of education and experience.	The International journal of social psychiatry	English	Observational	ACCESS Open Minds/Esprits Ouverts,	Canada	High-income North America	High SDI
(Markham et al, 2003)	The effects of the psychiatric label 'borderline personality disorder' on nursing staffs perceptions and causal attributions for challenging behaviours	British journal of clinical psychology	English	Observational	Psychology Department, South Warwickshire Primary Care Trust and University of Birmingham	UK, England	Western Europe	High SDI

(Martensson et al, 2014)	Mental health nursing staff's attitudes towards mental illness: an analysis of related factors	Journal of psychiatric and mental health nursing	English	Observational	University of Gävle. Faculty of Health and Occupational Studies, Department of Health and Caring Sciences	Sweden	Western Europe	High SDI
(Martin et al, 2019)	Attitudes to psychiatry and to mental illness among nursing students: Adaptation and use of two validated instruments in preclinical education	Journal of psychiatric and mental health nursing	English	Observational	Child Study Center, Yale School of Medicine	USA	High-income North America	High SDI
(Martinez-Martinez et al, 2019)	Effectiveness of direct contact intervention with people with mental illness to reduce stigma in nursing students	International journal of mental health nursing	English	Intervention	Universidad Europea de Valencia. Faculty of Health Sciences, Department of Nursing and Physiotherapy,	Spain	Western Europe	High SDI
(Mason et al, 2010)	Forensic nurses' perceptions of labels of mental illness and personality disorder: clinical versus management issues	Journal of psychiatric and mental health nursing	English	Observational	University of Chester. Faculty of Health and Social Care	UK, England	Western Europe	High SDI
(Mavundla et al, 1997)	The attitudes of nurses towards mentally ill people in a general hospital setting in Durban.	Curationis	English	Observational	University of Transkei. Department of Nursing Science	South Africa	Southern sub-Saharan Africa	Middle SDI
(Mavundla, 2000)	Professional nurses' perception of nursing mentally ill people in a general hospital setting	Journal of advanced nursing	English	Observational	Centre for Institutional Excellence and Research, Technikon SA	South Africa	Southern sub-Saharan Africa	Middle SDI
(McCarthy et al, 1973)	Nurses attitudes and expectations toward psychiatric patients with multiple readmissions	Nursing research	English	Observational	Boston City Hospital School of Nursing	USA	High-income North America	High SDI
(McConachie et al, 2009)	Mental health nurses' attitudes towards severe perinatal mental illness	Journal of advanced nursing	English	Observational	NHS Forth Valley. Department of Liaison Psychiatry	UK, Scotland	Western Europe	High SDI

(McLaughli, 1997)	The effect of classroom theory and contact with patients on the attitudes of student nurses towards mentally ill people	Journal of advanced nursing	English	Observational	Western Area College of Nursing, Multi-Disciplinary Education Centre, Altnagelvin Area Hospital	UK, Ireland	Western Europe	High SDI
(Moore et al, 1978)	Intercorrelations among factors in opinions about mental-illness scale in scores of non-psychiatric nurses - comparison with other studies	Psychological reports	English	Observational	Wayne State University, College of Nursing	USA	High-income North America	High SDI
(Morgan, 2016)	No Right Place to Die: Nursing Attitudes and Needs in Caring for People With Serious Mental Illness at End-of-Life	Journal of the american psychiatric nurses association	English	Observational	University of Massachusetts	USA	High-income North America	High SDI
(Morrison et al, 1976)	The attitudes of nursing students and others about mental illness.	Journal of psychiatric nursing and mental health services	English	Observational	Albany Medical College	USA	High-income North America	High SDI
(Morrison, 2009)	Using an adapted reflecting team approach to learn about mental health and illness with general nursing students: An Australian example	International journal of mental health nursing	English	Intervention	University of Canberra. Healthpact Research Centre for Health Promotion and Wellbeing, , ACT	Australia	Australasia	High SDI
(Mukesh et al, 2017)	Attitude towards mental illness among staff nurses in a tertiary care hospital-a hospital-based cross-sectional study	Journal of evolution of medical and dental sciences-jemds	English	Observational	Department of Psychiatry, Academy of Medical Sciences, Pariyaram, Kannur	India	South Asia	Low-middle SDI
(Munro et al, 2007)	Surveying the attitudes of acute mental health nurses	Journal of psychiatric and mental health nursing	English	Observational	The University of Manchester	UK, England	Western Europe	High SDI

(Napoletano, 1981)	Correlates of change in attitudes toward mental-illness among vocational nursing-students	Psychological reports	English	Observational	University of Colorado	USA	High-income North America	High SDI
(Ng et al, 2017)	Determining the effectiveness of a video-based contact intervention in improving attitudes of Penang primary care nurses towards people with mental illness	Plos one	English	Intervention	Department of Psychiatry, Penang Medical College	Malaysia	Southeast Asia	Middle SDI
(Ng, et al, 2010)	Growing practice specialists in mental health: addressing stigma and recruitment with a nursing residency program.	Nursing leadership	English	Intervention	Principal, Vision & Results Inc.	Canada	High-income North America	High SDI
(Nilsen et al, 2013)	Identifying Medical-Surgical Nursing Staff Perceptions of the Drug-Abusing Patient	Journal of addictions nursing	English	Observational	Naval Medical Center San Diego	USA	High-income North America	High SDI
(O'Ferrall-Gonzalez et al, 2019)	Factors associated with the evolution of attitudes towards mental illness in a cohort of nursing students	Journal of psychiatric and mental health nursing	English	Observational	Department of Nursing and Physiotherapy, University of Cádiz	Spain	Western Europe	High SDI
(Olade, 1979)	Attitudes towards mental-illness - comparison of post-basic nursing-students with science students	Journal of advanced nursing	English	Observational	University of Ibadan. College of Medicine, Department of Nursing	Nigeria	Western sub-Saharan Africa	Low-middle SDI
(Olade, 1983)	Attitudes towards mental-illness - effect of integration of mental-health concepts into a postbasic nursing degree program	Journal of advanced nursing	English	Observational	University of Ibadan. College of Medicine, Department of Nursing	Nigeria	Western sub-Saharan Africa	Low-middle SDI
(Ordan et al, 1974)	Nurses' professional stigma and attitudes towards postpartum women with severe mental illness	Journal of clinical nursing	English	Observational	Henrietta Szold/Hadassah-Hebrew University. School of Nursing, Faculty of	Israel	Western Europe	High SDI

					Medicine			
(Park et al, 2015)	Knowledge and Attitude of 851 Nursing Personnel toward Depression in General Hospitals of Korea	Journal of korean medical science	English	Observational	Department of Psychiatry, Yong-In Mental Hospital	South Korea	High-income Asia Pacific	High SDI
(Parra López et al, 2017)	Effects of clinical experience in the nursing student attitudes toward people with psychiatric disabilities	Ciencia y enfermería	Spanish	Intervention	Universidad Andrés Bello. Concepción. Enfermera, Mag. en Investigación Social y Desarrollo. Docente Facultad de Enfermería,	Chile	Southern Latin America	High-middle SDI
(Perlman et al, 2019)	Stigmatization Behavior of Pre-Registration Nurses: Do the Self-Determined Psychological Needs Influence This?	Issues in mental health nursing	English	Observational	University of Wollongong, School of Education	Australia	Australasia	High SDI
(Peterson, 2017)	Experiencing stigma as a nurse with mental illness	Journal of psychiatric and mental health nursing	English	Observational	Coast Mental Health, Rehabilitation & Recovery Program, Coquitlam	Canada	High-income North America	High SDI
(Phillips et al, 2019)	Practices and Attitudes of Nursing Students Toward Patients With Disordered Eating Behaviors.	Nursing education perspectives	Portuguese	Observational	Fairfield University. Egan School of Nursing and Health Studies	Portugal	Western Europe	High SDI
(Pinto-Foltz, 2009)	Reducing Stigma Related to Mental Disorders: Initiatives, Interventions, and Recommendations for Nursing	Archives of psychiatric nursing	English	Review	University of Louisville. School of Nursing	USA	High-income North America	High SDI

(Poreddi et al, 2014)	Undergraduate Nursing Students' Attitudes towards Mental Illness: Implications for Specific Academic Education.	Indian journal of psychological medicine	English	Observational	Deemed University. Department of Nursing, College of Nursing, National Institute of Mental Health and Neuro Sciences	India	South Asia	Low-middle SDI
(Poreddi et al, 2015)	Bachelor of nursing student' attitude towards people with mental illness and career choices in psychiatric nursing. An Indian perspective.	Investigacion y educacion en enfermeria	English	Observational	Deemed University. Department of Nursing, National Institute of Mental health and Neurosciences	India	South Asia	Low-middle SDI
(Poreddi et al, 2017)	Medical and nursing students' attitudes toward mental illness: An Indian perspective.	Investigacion y educacion en enfermeria	English	Observational	Deemed University. Department of Nursing, National Institute of Mental health and Neurosciences	India	South Asia	Low-middle SDI
(Reed et al, 2005)	The mixed attitudes of nurse's to caring for people with mental illness in a rural general hospital	International journal of mental health nursing	English	Observational	Maldon Hospital	Australia	Australasia	High SDI
(Robinson, 1973)	Registered and student psychiatric nurses opinions about mental-illness	New zealand medical journal	English	Observational	Oakley Hosp,Mental Hlth Res Fdn,Auckland	New Zealand	Australasia	High SDI
(Rodriguez-Almagro et al, 2019)	Level of Stigma among Spanish Nursing Students toward Mental Illness and Associated Factors: A Mixed-Methods Study	International journal of environmental research and public health	English	Observational	University of Castilla-La Mancha. Department of Nursing, Ciudad Real Nursing Faculty	Spain	Western Europe	High SDI
(Ross et al, 2009)	Stigma, negative attitudes and discrimination towards mental illness within the nursing profession: a review of the literature	Journal of psychiatric and mental health nursing	English	Review	Douglas College, BSN Program, Faculty of Health Sciences	Canada	High-income North America	High SDI

(Rydon, 2005)	The attitudes, knowledge and skills needed in mental health nurses: The perspective of users of mental health services	International journal of mental health nursing	English	Observational	Massey University. School of Health Sciences, Albany Campus,	New Zealand	Australasia	High SDI
(Sahile et al, 2019)	Primary health care nurses attitude towards people with severe mental disorders in Addis Ababa, Ethiopia: a cross sectional study	International journal of mental health systems	English	Observational	Amanuel Mental Specialized Hospital, Addis Ababa	Ethiopia	Eastern sub-Saharan Africa	Low SDI
(Samari et al, 2018)	Stigma towards people with mental disorders: Perspectives of nursing students	Archives of psychiatric nursing	English	Observational	Research Division, Institute of Mental Health	Singapore	High-income Asia Pacific	High SDI
(Samari et al, 2019)	Attitudes towards psychiatry amongst medical and nursing students in Singapore	Bmc medical education	English	Observational	Research Division, Institute of Mental Health, Buangkok Green Medical Park	Singapore	High-income Asia Pacific	High SDI
(Sari et al, 2018)	Investigation of attitudes toward mental illness among nursing students in Indonesia	International journal of nursing sciences	English	Observational	Diponegoro University. School of Nursing, Faculty of Medicine	Indonesia	Southeast Asia	Middle SDI
(Schafer et al, 2011)	A survey into student nurses' attitudes towards mental illness: Implications for nurse training	nurse education today	English	Observational	Anglia Ruskin Univ, Chelmsford	UK, England	Western Europe	High SDI
(Seo Moon-sook, 2017)	A Subjectivity Study on the Attitudes toward Mental Disorders of Nursing Students	The Journal of Korean Nursing Research	Korean	Observational	McMaster University. Department of Health Reserach Methods Evidence and Impact (HEI)	Canada	High-income North America	High SDI

(Sercu et al, 2015)	How does stigma influence mental health nursing identities? An ethnographic study of the meaning of stigma for nursing role identities in two Belgian Psychiatric Hospitals	International journal of nursing studies	English	Observational	Ghent University. Department of Sociology, Research Foundation (FWO)	Belgium	Western Europe	High SDI
(Shahif et al, 2019)	Knowledge and attitude towards mental illness among primary healthcare nurses in Brunei: A cross-sectional study	Asian journal of psychiatry	English	Observational	University of Brunei Darussalam. PAPRSB Institute of Health Science	Brunei	High-income Asia Pacific	High SDI
(Shattell et al, 2006)	She took the time to make sure she understood: Mental health patients' experiences of being understood	Archives of psychiatric nursing	English	Observational	University of North Carolina at Greensboro. School of Nursing	USA	High-income North America	High SDI
(Sherwood, 2019)	Healthcare curriculum influences on stigma towards mental illness: Core psychiatry course impact on pharmacy, nursing and social work student attitudes	Currents in pharmacy teaching and learning	English	Observational	University of New England. College of Pharmacy	USA	High-income North America	High SDI
(Slemon et al, 2019)	Undergraduate students' perspectives on pursuing a career in mental health nursing following practicum experience	Journal of clinical nursing	English	Observational	University of British Columbia. School of Nursing	Canada	High-income North America	High SDI
(Slimmer et al, 1990)	Effect of psychiatric clinical learning site on nursing students' attitudes toward mental illness and psychiatric nursing.	The Journal of nursing education	English	Intervention	University of Illinois System	USA	High-income North America	High SDI
(Smith, 1977)	Attitudes of student psychiatric nurses towards mental illness.	Nursing times	English	Observational	City University. Research Fellow, Department of Mental Health and Learning	UK, England	Western Europe	High SDI

					Disability Nursing			
(Smyth et al, 1971)	Effects of nursing training on attitudes toward psychiatric patients	Psychological reports	English	Observational	University of Tokyo. Graduate School of Arts and Sciences	Japan	High-income Asia Pacific	High SDI
(Song et al, 2016)	Experience Process of Reducing Prejudices against People with Mental Illness by Nursing Students during Clinical Training in Psychiatric Wards	Journal of Korean Academy of Psychiatric and Mental Health Nursing	Korean	Observational	National Naju Hospital	South Korea	High-income Asia Pacific	High SDI
(Song, 2015)	The Nursing Students' Experience of Psychiatric Practice in South Korea	Archives of psychiatric nursing	English	Observational	Wonkwang University. Department of Nursing, Center for Animal Resources Development	South Korea	High-income Asia Pacific	High SDI
(Soyoung et al, 2016)	Social Distance Attitudes of Nursing Students towards Adults with Mental Disorders	Journal of Korean Academy of Psychiatric and Mental Health Nursing	Korean	Observational	Kyung Hee University.College of Nursing Science	South Korea	High-income Asia Pacific	High SDI
(Sreeraj et al, 2017)	Indian nursing students' attitudes toward mental illness and persons with mental illness.	Industrial psychiatry journal	English	Observational	Department of Psychiatry, National Institute of Mental Health and Neurosciences	India	South Asia	Low-middle SDI
(Sreevani et al, 2012)	Assessment of the attitude of staff nurses towards hospitalised psychiatric patients in Kolar (Karnataka).	The Nursing journal of India	English	Observational	Reade, Centre for Rehabilitation and Reumatology	Netherlands	Western Europe	High SDI

(Stacey et al, 2018)	A focus group study exploring student nurse's experiences of an educational intervention focused on working with people with a diagnosis of personality disorder	Journal of psychiatric and mental health nursing	English	Intervention	University of Nottingham. School of Health Sciences, Royal Derby Hospital	UK, England	Western Europe	High SDI
(Stewart et al, 2015)	Thematic analysis of psychiatric patients' perceptions of nursing staff	International journal of mental health nursing	English	Observational	Kings College. Institute of Psychiatry	UK, England	Western Europe	High SDI
(Stone, 1966)	Change in nursing students expectations regarding mental patients	Nursing research	English	Observational	University of North Dakota. Department of Community Medicine,	USA	High-income North America	High SDI
(Stuhlmiller et al, 2019)	Understanding the impact of mental health placements on student nurses' attitudes towards mental illness	Nurse education in practice	English	Observational	University at Buffalo, School of Nursing	USA	High-income North America	High SDI
(Sun et al, 2014)	Attitudes towards people with mental illness among psychiatrists, psychiatric nurses, involved family members and the general population in a large city in Guangzhou, China	International journal of mental health systems	English	Observational	Guangzhou Psychiatric Hospital. Neuropsychiatric Research Institute	China	East Asia	High-middle SDI
(Surgenor et al, 2005)	Nursing student attitudes to psychiatric nursing and psychiatric disorders in New Zealand	International journal of mental health nursing	English	Observational	Department of Psychological Medicine, Christchurch School of Medicine and Health Sciences	New Zealand	Australasia	High SDI
(Swain et al, 1973)	Nursing students attitudes toward mental illness	Nursing research	English	Observational	Univ Wisconsin,Sch Nursing	USA	High-income North America	High SDI

(Tay et al, 1976)	Nurses' attitudes toward people with mental illnesses in a psychiatric hospital in Singapore.	Journal of psychosocial nursing and mental health services	English	Observational	Nursing Department, Institute of Mental Health, Woodbridge Hospital	Singapore	High-income Asia Pacific	High SDI
(Tei-Tominaga et al, 2014)	Stigma towards nurses with mental illnesses: A study of nurses and nurse managers in hospitals in Japan	International journal of mental health nursing	English	Observational	Kyoto Tachibana University. Faculty of Nursing	Japan	High-income Asia Pacific	High SDI
(Thongpriwan et al, 2015)	Undergraduate nursing students' attitudes toward mental health nursing	Nurse education today	English	Observational	University of Wisconsin-Milwaukee	USA	High-income North America	High SDI
(Vaghee et al, 2017)	Comparing the Effect of Contact-based Education with Acceptance and Commitment Training on Destigmatization Toward Psychiatric Disorders in Nursing Students	Iranian journal of psychiatry and behavioral sciences	English	Intervention	Mashhad University of Medical Sciences. School of Nursing and Midwifery, Evidence Based Care Research Centre	Iran	North Africa and Middle East	Middle SDI
(van der Kluit et al, 2011)	Factors influencing attitudes of nurses in general health care toward patients with comorbid mental illness: an integrative literature review.	Issues in mental health nursing	English	Review	Reade, Centre for Rehabilitation and Reumatology	Netherlands	Western Europe	High SDI
(Vijayalakshmi et al, 2013)	Attitudes of undergraduates towards mental illness: A comparison between nursing and business management students in India	South african journal of psychiatry	English	Observational	National Institute of Mental Health and Neuro Sciences	India	South Asia	Low-middle SDI

(Walsh, 1971)	Instruction in psychiatric nursing, level of anxiety, and direction of attitude change toward mentally ill	Nursing research	English	Observational	Natl League Nursing,Dept Baccalaureate & Higher Degree Programs	USA	High-income North America	High SDI
(Weare et al, 2019)	ICU nurses feel unprepared to care for patients with mental illness: A survey of nurses' attitudes, knowledge, and skills	Intensive and critical care nursing	English	Observational	Peninsula Hlth, Dept Intens Care	Australia	Australasia	High SDI
(Webb et al, 2007)	Nursing staff attitudes towards patients with personality disorder	Personality and mental health	English	Observational	Cardiff University. The Gwylfa Therapy Service, St Cadoc's Hospital and School of Psychology	UK, Wales	Western Europe	High SDI
(Webster, 2009)	Addressing Nursing Students' Stigmatizing Beliefs Toward Mental Illness	Journal of psychosocial nursing and mental health services	English	Intervention	Salisbury University Depatment of Nursing	USA	High-income North America	High SDI
(Weller et al, 1988)	Does contact with the mentally-ill affect nurses attitudes to mental-illness	British journal of medical psychology	English	Observational	Bar-Ilan University. Department of Sociology	Israel	Western Europe	High SDI
(Westwood et al, 2010)	Attitudes and perceptions of mental health nurses towards borderline personality disorder clients in acute mental health settings: a review of the literature	Journal of psychiatric and mental health nursing	English	Review	University of Manchester. Nursing Midwifery and Social Work	UK, England	Western Europe	High SDI
(Wilkinson, 1982)	The effects of brief psychiatric-training on the attitudes of general nursing-students to psychiatric-patients	Journal of advanced nursing	English	Intervention	University of Kent. School of Psychology	UK, England	Western Europe	High SDI

(Winkler et al, 2017)	Short video interventions to reduce mental health stigma: a multi-centre randomised controlled trial in nursing high schools	Social psychiatry and psychiatric epidemiology	English	Intervention	Department of Social Psychiatry, National Institute of Mental Health	Czech Republic	Central Europe	High-middle SDI
(Yamauchi et al, 2011)	Effects of psychiatric training on nursing students' attitudes towards people with mental illness in Japan	International journal of social psychiatry	English	Observational	University of Tokyo. Graduate School of Arts and Sciences	Japan	High-income Asia Pacific	High SDI
(Yoon et al, 2007)	A Study on the Difference of Practicum Satisfaction and Attitudes toward Mental Disorder between Nursing Students and Social Welfare Students Who Experienced Psychiatric Practicum.	Journal of Rehabilitation Research	Korean	Observational	Gyeongju University	South Korea	High-income Asia Pacific	High SDI
(Younghee, 2018)	Effects of Mental health clinical practice on the attitude toward the mental illness, social distance, psychiatry nurses' image of Nursing Students	Journal of the Korea Convergence Society	Korean	Observational	Kwangju Women's University. Dept. of Nursing	South Korea	High-income Asia Pacific	High SDI
(Yuan, et al, 2018)	Direct and moderating effects of personality on stigma towards mental illness	BMC psychiatry	English	Observational	Research Division, Institute of Mental Health	Singapore	High-income Asia Pacific	High SDI
Harrison et al, 2017)	Breaking down the stigma of mental health nursing: A qualitative study reflecting opinions from western australian nurses	Journal of psychiatric and mental health nursing	English	Observational	University of Western Australia, School of Psychiatry and Clinical Neurosciences	Australia	Australasia	High SDI

†GBD Regions; Global Burden of Disease Regions

†† Source: http://ghdx.healthdata.org/record/ihme-data/gbd-2019-socio-demographic-index-sdi-1950-2019

Legend

SDI quintile	Lower bound	Upper bound
Low SDI	0	0,454743
Low-middle SDI	0,454743	0,607679
Middle SDI	0,607679	0,689504
High-middle SDI	0,689504	0,805129
High SDI	0,805129	1
